# Awareness screening and referral patterns among pediatricians in the United States related to early clinical features of spinal muscular atrophy (SMA)

**DOI:** 10.1186/s12887-021-02692-2

**Published:** 2021-05-17

**Authors:** Mary Curry, Rosángel Cruz, Lisa Belter, Mary Schroth, Megan Lenz, Jill Jarecki

**Affiliations:** Cure SMA, 925 Busse Road, Elk Grove Village, IL 60007 USA

## Abstract

**Background:**

Spinal Muscular Atrophy (SMA), a leading genetic cause of death in infants, is an autosomal recessive neuromuscular disease characterized by progressive muscle weakness and atrophy. While early diagnosis of SMA is critical to modifying disease progression and improving outcomes, serious diagnostic delays persist. There is a need to improve SMA awareness, screening, and referral patterns.

**Methods:**

Two online surveys, developed by Cure SMA for general pediatricians, were distributed by Medscape Education via email (September 2018, *n = 300*, December 2019, *n = 600*). The surveys asked about adherence to the American Academy of Pediatrics (AAP) developmental screening and surveillance guidelines, comfort with identification of early signs of neuromuscular disease (NMD), familiarity with SMA, and barriers to timely referral.

**Results:**

In 2018, 70.3% of survey respondents indicated comfort in identifying early signs of NMD and 67.3% noted familiarity with SMA. 52.7% correctly indicated the need for genetic testing to make a definitive diagnosis of SMA, 74.0% meet or exceed developmental screening recommendations, and 52.0% said they would immediately refer to a specialist. In 2019, with a larger sample, 73.0% adhere to developmental screening guidelines, and awareness of the genetic testing requirement for SMA was significantly lower by 7.7% (*p* < 0.03). Specialist wait times emerged as a barrier to referral, with 64.2% of respondents citing wait times of 1–6 months.

**Conclusions:**

Many pediatricians underutilize developmental screening tools and lack familiarity with diagnostic requirements for SMA. Continuing efforts to expand awareness and remove barriers to timely referral to SMA specialists, including reducing appointment wait times, are needed.

**Supplementary Information:**

The online version contains supplementary material available at 10.1186/s12887-021-02692-2.

## Introduction

SMA is an autosomal recessive neuromuscular disorder (NMD) characterized by progressive muscle weakness and atrophy [1–5]. With an incidence rate of approximately 1 in 11,000 newborns, SMA was the number-one monogenetic cause of death for infants prior to the FDA approval of disease-modifying treatments [6, 7]. SMA is typically classified into four types based on severity and age of symptom onset [5, 8–15]. SMA Type I being the most severe and common form of the disease [5, 8–15]. In infants with SMA Type I, the onset of irreversible denervation occurs within the first 3 months, with loss of 90% of motor units occurring by 6 months of age [16, 17]. Prior to treatment, these infants were unable to sit independently and typically required invasive supportive care with the progression of symptoms [5, 8, 9]. SMA Type II is typically diagnosed after 6 months of age, but before 2 years of age, and although many achieve independent sitting, infants are historically unable to walk and even stand [5, 9, 10]. SMA Type III is usually diagnosed in children after 18 months of age, but before 3 years of age [5, 9, 10]. Affected individuals are initially able to walk, but have increasingly limited mobility overtime [5, 9, 10]. Type IV is a rare form of SMA; symptoms appear in adulthood as mild proximal muscle weakness [5, 10].

Until recently, there were no disease-modifying treatments for SMA. Discoveries about the genetic mechanisms and pathophysiology of SMA spurred efforts to develop disease modifying drug and gene-based treatments aimed at slowing the progression of the disease, culminating in the landmark US Food and Drug Administration (FDA) approvals of nusinersen (an antisense oligonucleotide) in 2016 [18], onasemnogene abeparvovec-xioi (gene-replacement therapy) in 2019 [19], and risdiplam (survival of motor neuron 2 (*SMN2*) splicing modifier) in 2020 [20]. Clinical trials have demonstrated that early treatment is critical to modifying disease progression while improving health outcomes and life expectancy for patients with SMA [21–23]. Clinical trial data and real-world evidence continue to support the critical correlation between early administration of treatment and maximum opportunity for improved outcomes for patients [21–28].

Pediatric neuromuscular disorders occurring during infancy, such as SMA, often present with hypotonia, weakness, and absent reflexes [29]. Assessment of developmental delay assists with the identification of early key concomitant signs of NMDs, including difficulty swallowing during feedings, failure to thrive, and early gross motor delays such as head lag and slowed movement of limbs when supine [29, 30]. The combination of symptoms and age of onset helps to narrow the differential diagnosis of neuromuscular disorders; a clear clinical picture is required to expedite appropriate evaluation of symptoms and obtain an early and accurate diagnosis [29, 30].

Although early diagnosis and treatment is vital to allow for effective interventions before severe permanent neuron damage occurs, significant diagnostic delay for SMA patients persists [31, 32]. Recent literature reviews indicate that SMA Type I infants are not diagnosed until the mean age of 6.3 months (which overlaps with a period of denervation characteristic of the most severe form of the disease), despite findings that average symptom onset occurs at approximately 2.5 months [31, 32].

In 2018, SMA was added to the federal Recommended Uniform Screening Panel (RUSP) for newborn screening [33]. As of this writing, only 34 states have adopted the RUSP recommendation for inclusion of SMA in newborn screening, with 68% of newborns receiving screening for SMA [34]. Despite significant progress, the lack of universal adoption of newborn screening represents a missed opportunity for early diagnosis. Additionally, given the complexity of the pediatric-specialty care interface, including delivery deficiencies, busy neurologists’ schedules, and lack of adequate health insurance, interference in the delivery of effective care coordination and referral may occur [35, 36]. Families also often cite frustrating diagnostic odysseys as they consult various physicians to rule out potential conditions and pinpoint a firm diagnosis of SMA [31, 37].

To better understand the underlying determinants for delay to SMA diagnosis and identify barriers that can be addressed, in 2018 and 2019 Cure SMA conducted two landmark surveys among pediatricians. The first survey aimed to evaluate awareness and familiarity with SMA among physicians while the follow-up survey, 1 year later, expanded the focus to include assessment of pediatricians’ adherence to the American Academy of Pediatrics (AAP) developmental screening and surveillance guidelines [38–40], and referral patterns. These surveys provide foundational information to support Cure SMA’s ongoing SMArt Moves initiative [41], a disease awareness and educational campaign launched in 2018 to empower parents and healthcare professionals to promptly recognize and diagnose the early signs of SMA.

## Methods

The 2018 survey included 11 questions that focused on awareness of SMA, diagnostic requirements for the disorder, and developmental screening tool utilization (refer to Additional file 1: Appendix 1). The 2019 survey comprised 27 questions seeking detailed practice information and information about patterns and barriers to specialist referrals (refer to Additional file 1: Appendix 2). Each study qualified as exempt research by Western Institutional Review Board (WIRB).

Both surveys were distributed via Survey Monkey to a large database of pediatricians in the United States (21,264 pediatricians were contacted between September 19, 2018 and September 28, 2018 and 19,096 were contacted between December 3, 2019 and January 2, 2020) in partnership with Medscape Education.

Descriptive statistics were calculated for all survey variables, and chi-square tests were conducted to test associations between categorical variables from the 2018 and 2019 survey. A binomial logistic regression was used to predict whether a pediatrician will immediately refer an infant or toddler to a pediatric neurologist for further evaluation based on their comfort level of recognizing early signs of a neuromuscular disease.

The 2019 survey included three questions (with slight text variations) similar to ones that were previously asked in the 2018 survey, allowing for comparison across a one-year timeframe. These questions focused on assessment of which developmental screening tools were used in a provider’s practice, how frequently those tools were administered, and the required procedures to make a definitive diagnosis of SMA.

## Results

A total of 300 pediatricians completed the first survey in September 2018. The second survey received 600 completed responses in December 2019 (through January 2, 2020). The overall response rate was 2.3% in 2018 and 4.5% in late 2019 (Table 1). Available demographic information, including information about respondents’ years in practice (for both surveys) and detailed practice information (available for the 2019 survey only), appears in Tables 2 and 3, respectively. A subset of 42 individuals completed the survey in both years. Amongst this subset of participants there was high consistency between the two survey years for the years in practice, κ = .78, *p* < .0001, and fair consistency between the two survey years for identifying genetic testing being the correct method of SMA diagnosis, κ = .30, *p* = 0.02.

**Table 1:** 2018 and 2019 Surveys Response Data

	2018 Ped Survey	2019 Ped Survey
Total Invited	21,264	19,095
Completed	300	600
Disqualified	40	138
Dropout	150	123
Response Rate	2.30%	4.50%
Average # times contacted	2	3

Eligibility was determined by provider specialty; those that did not self-identify as general pediatricians were ‘Disqualified’ from participation. Additionally, individuals that partially completed the survey were reported as a ‘Dropout’

**Table 2:** 2018 & 2019 Survey Participant Demographics

How many years have you been in practice?	2018 Responses	2019 Responses	***P*** value
0–10 years	40.7% (122)	32.7% (196)	0.04
11–20 years	29.0% (87)	31.5% (189)	
21–30 years	20.7% (62)	21.0% (126)	
Over 30 years	9.7% (29)	14.8% (89)

**Table 3:** 2019 Participant Demographics

**Average No. of Pts Seen Weekly**	
0 to 10	2.0% (12)
11 to 25	5.2% (31)
26 to 50	15.3% (92)
51 to 75	25.3% (152)
> 75	52.2% (313)
**Practice Location**	
Urban	38.2% (229)
Rural	10.8% (65)
Suburban	51.0% (306)
**Practice Type**	
Solo practice	9.7% (58)
Single specialty group	44.2% (265)
Multi-specialty group	22.2% (133)
Direct hospital employee/ contractor	9.0% (54)
Academic faculty practice	11.7% (70)
Other	3.3% (20)
**No. of Physicians in Practice**	
Solo practice	9.7% (58)
2 to 4	26.0% (156)
5 to 10	31.7% (190)
11 to 24	14.0% (84)
25 to 49	5.5% (33)
50+	13.2% (79)
**No. of Managed Care Contracts**	
0	10.0% (60)
1 to 4	37.5% (225)
5 to 9	29.7% (178)
10+	22.8% (137)

The 2018 survey indicated a lack of awareness about the diagnostic requirements for SMA. 52.7% correctly indicated that genetic testing is required to make a definitive diagnosis of SMA, while 31.0% chose muscle biopsy.

In the 2019 survey, responses indicate a persistent lack of awareness of the diagnostic requirements for SMA. Additionally, a comparison between responses from each survey revealed a decrease in the percent of providers correctly identifying use of genetic testing to obtain a definitive SMA diagnosis (52.7% in 2018 vs. 45.0% in 2019, *p* < 0.03) (Table 4).

**Table 4:** 2018 and 2019 Surveys Comparison of Tests Required for SMA Diagnosis

	Each test comparison		
	2018 Response	2019 Response	***p*** value
**Electromyography**			0.189
Yes	11.3%	14.5%	
No	88.7%	85.5%	
**Genetic Testing**			0.03
Yes	52.7%	45.0%	
No	47.3%	55.0%	
**Muscle Biopsy**			0.068
Yes	31.0%	37.2%	
No	69.0%	62.8%	
**Serum Creatinine Kinase**			0.017
Yes	4.3%	1.7%	
No	95.7%	98.3%	
**Other**			0.218
Yes	0.7%	1.7%	
No	99.3%	98.3%	

When asked in 2018 how screening tools are utilized in clinic, respondents were provided an opportunity to select all conditions that apply within their practice. 56.0% of respondents indicated use at each well visit. Additionally, 41.3% utilized screening tools at the 9-, 18-, and 30-month well visits, 32.7% utilized screening tools as soon as concerns appear during developmental surveillance, and 18.0% indicated use at both time points (Table 5). However, a deeper analysis revealed that 22.7% of providers identified screening tool usage at the 9-, 18-, and 30-month well visits, but not as concerns appear during surveillance; also, 9.3% of respondents only utilize screening tools as concerns appear during developmental surveillance and not at well visits, as recommended by current guidelines.

**Table 5:** 2018–2019 Comparison of Utilization Frequency of Screening Tools in Clinic

	Each frequency comparison		
	2018 Response^**a**^	2019 Response	***p*** value
**Tools are administered as concerns appear during developmental surveillance**			< 0.0001
Yes	32.7%	8.0%	
No	67.3%	92.0%	
**Tools are administered at 9-, 18-, and 30-month visits**			< 0.0001
Yes	41.3%	15.0%	
No	58.7%	85.0%	
**Tools are administered at each well visit**			< 0.0001
Yes	56.0%	37.3%	
No	44.0%	62.7%	
**Tools are administered at 9-, 18-, and 30-month visits and as concerns appear during developmental surveillance**			< 0.0001
Yes	18.0%	35.7%	
No	82.0%	64.3%	
**Tools are not administered in practice**			Option not provided in 2018 Survey
Yes		4.0%	
No		96.0%	

In 2019, respondents were instructed to select the option that best described the frequency of tool administration within their practice. ^a^In 2018 each pediatrician had the option to ‘*select all that apply*’ for the following provided responses: Tools are administered as concerns appear during developmental surveillance; Tools are administered at 9-, 18-, and 30-month visits; Tools are administered at each well visit. Respondents that selected both ‘Tools are administered as concerns appear during developmental surveillance’ and ‘Tools are administered at 9-, 18-, and 30-month visits’ were also listed within the row labeled ‘Tools are administered at 9-, 18-, and 30-month visits and as concerns appear during developmental surveillance’

In the 2019 survey, responses indicate a persistent underutilization of developmental screening tools. For additional clarity, in the 2019 survey the framing of the question was revised. Respondents were asked to identify the condition that best describes screening tool utilization in their clinic. 37.3% of respondents indicated use at each well visit, and 35.7% indicated use at the 9-, 18-, and 30-month well visits and as concerns appear during developmental surveillance. However, 15.0% of providers only utilize screening tools at the 9-, 18-, and 30-month well visits, and do not also utilize screening tools as concerns appear during surveillance. Additionally, 8.0% of respondents only utilize screening tools as concerns appear during developmental surveillance and not at the well visits, as recommended by current guidelines. Among those surveyed, 4.0% reported not utilizing developmental screening tools in their practice (Table 5).

In addition, in 2019 when the frequency of screening tool utilization was examined for associations with participant demographics, providers that recently completed their training (those with 0 to 10 years of practice experience) were 1.549 times more likely to use tools at every well visit or at the 9-, 18-, and 30-month visit as compared with those with more than 10 years of practice experience.

Respondents were asked to indicate which tools, recommended by Bright Futures [40], that they utilize in clinic. When provided the option to ‘select all that apply’, 74.7% of providers in 2018 indicated usage of the Ages and Stages Questionnaire (ASQ-3) [42], while 29.3% utilize the Denver-II Developmental Screening Test [43], and the Parents’ Evaluation of Developmental Status (PEDS) [44] measures. When similarly asked in 2019, 67.5% of providers indicated usage of the ASQ-3 [42]. Additionally, 35.2% utilize the Denver-II Developmental Screening Test [43], and 32.7% utilize the PEDS [44] (Table 6).

**Table 6:** 2018 & 2019 Survey Comparison of Screening Tools

	2018 Response	2019 Response	***p*** value
**Ages and Stages Questionnaire (ASQ-3)**			0.027
Yes	74.7%	67.5%	
No	25.3%	32.5%	
**Battelle Developmental Inventory Screening (BDI-ST)**			0.01
Yes	0.7%	3.5%	
No	99.3%	96.5%	
**Bayley Infant Neurodevelopmental Screen (BINS)**			< 0.0001
Yes	10.7%	3.5%	
No	89.3%	96.5%	
**Child Development Inventory (CDI)**			0.4
Yes	11.7%	13.7%	
No	88.3%	86.3%	
**Child Development Review-Parent Questionnaire (CDR-PQ)**			0.642
Yes	9.7%	10.7%	
No	90.3%	89.3%	
**Denver-II Developmental Screening Test**			0.08
Yes	29.3%	35.2%	
No	70.7%	64.8%	
**Infant Development Inventory**			0.278
Yes	6.0%	8.0%	
No	94.0%	92.0%	
**Parents’ Evaluation of Developmental Status (PEDS)**			0.31
Yes	29.3%	32.7%	
No	70.7%	67.3%	

**Fig. 1:**
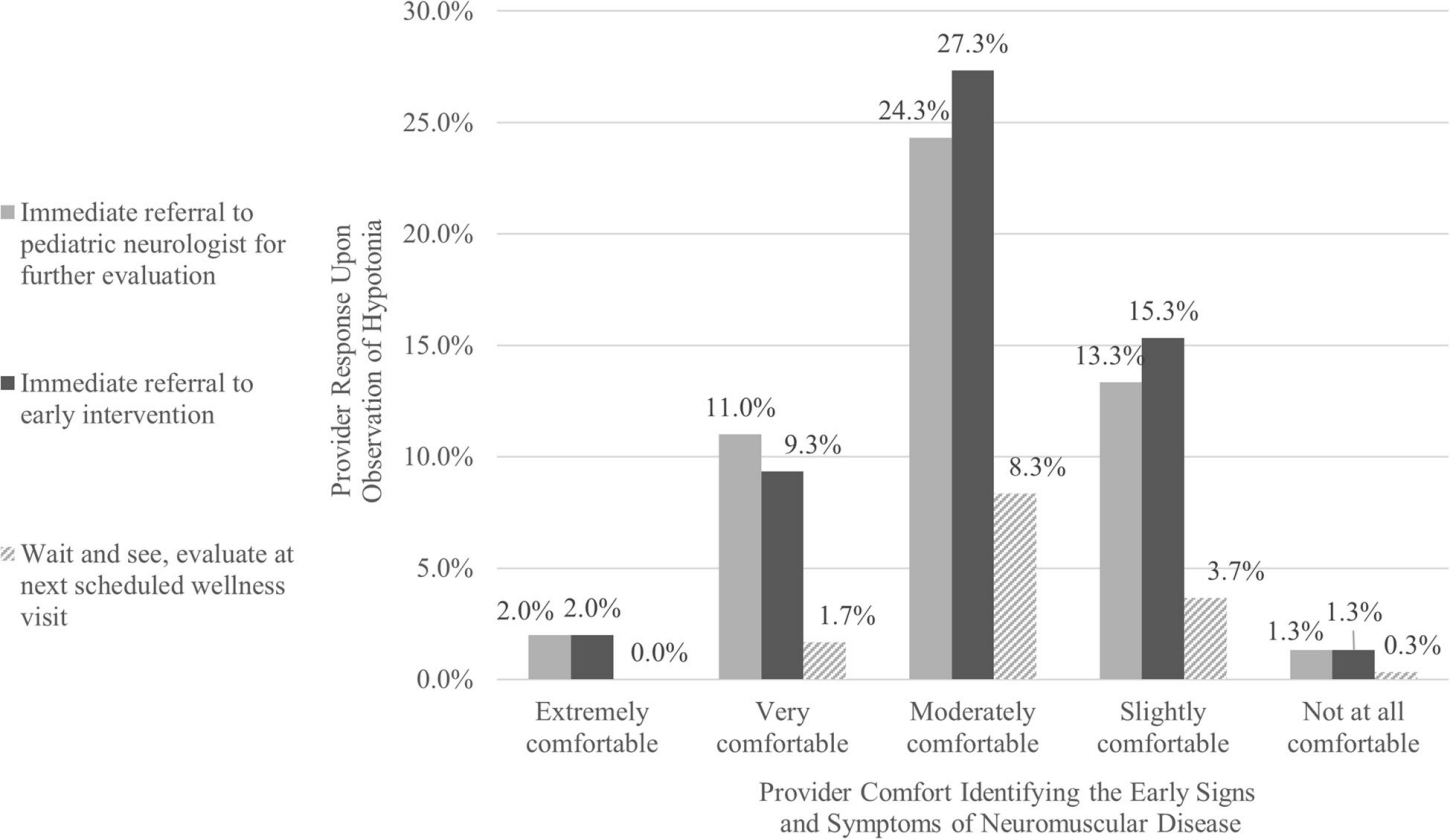
Providers’ Response Upon Observation of Hypotonia by Self-Reported Comfort Identifying Neuromuscular Disease (2018 Survey). The breakdown of self-reported comfort identifying the early signs of NMD is as follows: Extremely comfortable: 3.3% (*n* = 10); Very comfortable: 18.7% (*n* = 56); Moderately comfortable: 48.3% (*n* = 145); Slightly Comfortable: 26.7% (*n* = 80); Not at All Comfortable: 3.0% (*n* = 9). The breakdown of provider response upon observation of hypotonia in an infant or toddler* is as follows: Immediate referral to pediatric neurologist for further evaluation: 52.0% (*n* = 156); Immediate referral to early intervention for further evaluation: 55.3% (*n* = 166); Wait and see, evaluate at next scheduled well visit: 14.0% (*n* = 42).* Each pediatrician had the option to ‘select all that apply’ for the provided responses

**Fig. 2:**
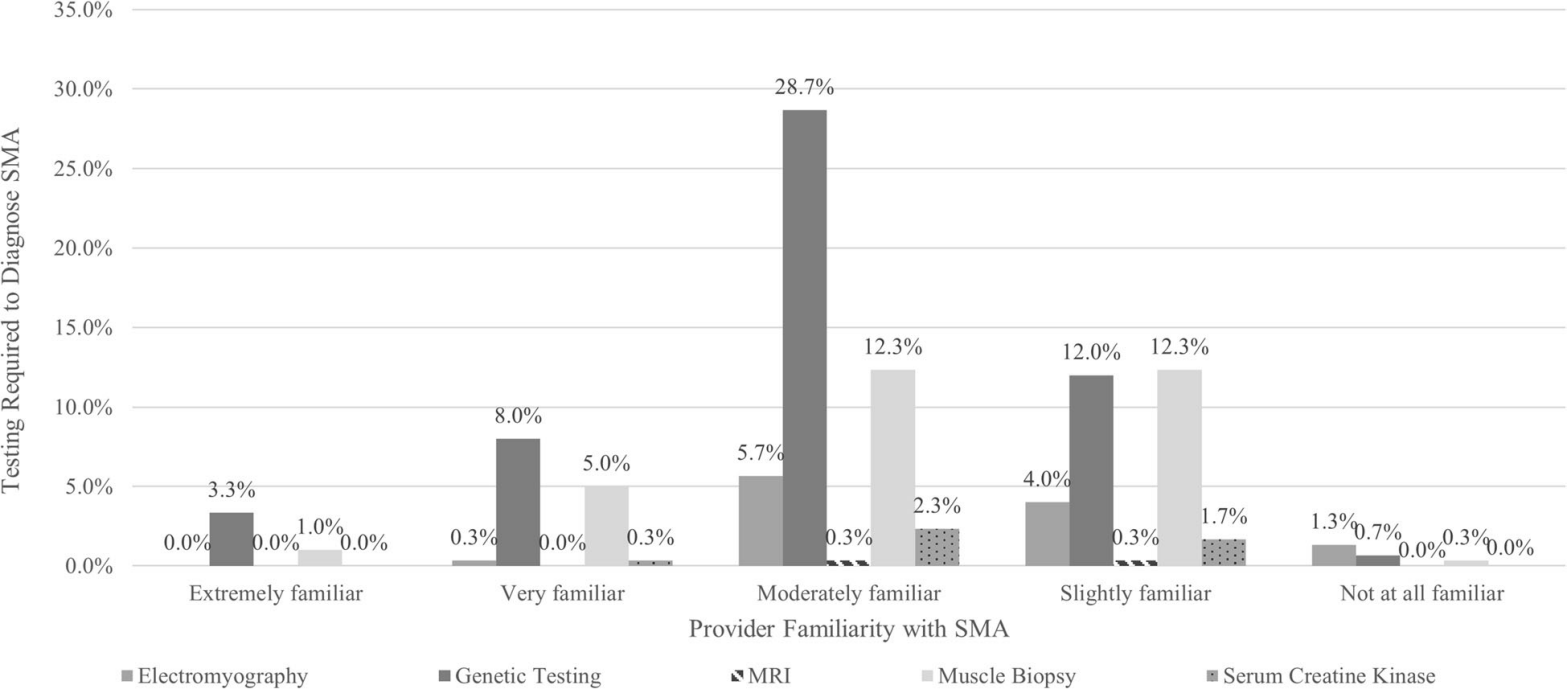
Provider Awareness of SMA Diagnostic Requirements Organized by Self-Reported Familiarity with the Disease (2018 Survey). The breakdown of self-reported familiarity with SMA is as follows: Extremely familiar: 4.3% (*n* = 13); Very familiar: 13.7% (*n* = 41); Moderately familiar: 49.3% (*n* = 148); Slightly Familiar: 30.3% (*n* = 91); Not at All Familiar: 2.3% (*n* = 7). The breakdown of provider response regarding the testing required for SMA diagnosis is as follows: Electromyography: 11.3% (*n* = 34); Genetic testing: 52.7% (*n* = 158); MRI: 0.7% (*n* = 2); Muscle biopsy: 31.0% (*n* = 93); Serum creatine kinase: 4.3% (*n* = 13)

Additional information from the 2018 survey indicated that, upon observation of hypotonia, 55.3% of pediatricians indicated they would immediately refer to early intervention, while 52.0% would immediately refer to a pediatric neurologist for further evaluation, 50.3% said they would schedule an early return visit (within a month), 14.0% would ‘wait and see’ or evaluate at the next scheduled well child visit, and 17.7% would order a serum creatinine kinase test (participants were given the option to select ‘all that apply’). Additionally, 70.3% of respondents indicated comfort identifying the early signs of NMD (‘Extremely comfortable’ 3.3%; ‘Very comfortable’ 18.7%; ‘Moderately comfortable’ 48.3%). When comparing the providers’ self-reported comfort in identifying the early signs and symptoms of NMD with responses for the question that assesses the providers’ typical course of action upon observation of hypotonia in an infant or toddler (Fig. 1), those reporting they were ‘Extremely comfortable’ or ‘Very comfortable’ were 1.47 times more likely to ‘immediately refer [the infant or toddler] to a pediatric neurologist for further evaluation’. Furthermore, 67.3% of respondents noted a familiarity with SMA (‘Extremely familiar’ 4.3%; ‘Very familiar’ 13.7%; ‘Moderately familiar’ 49.3%); however, only 59.4% of this group correctly identified the genetic testing requirement (Fig. 2).

**Fig. 3:**
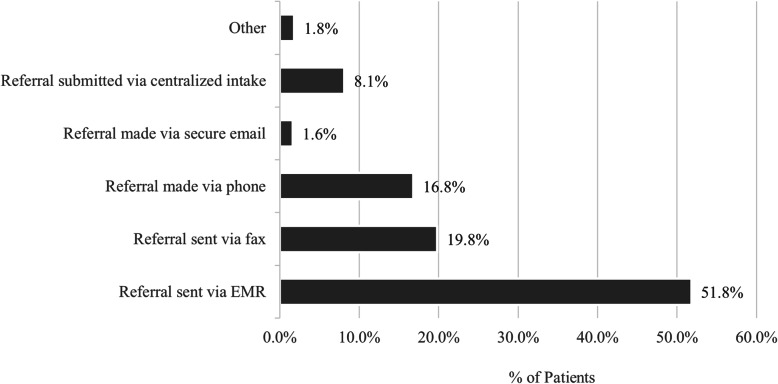
Percent (%) of patients referred via each method across all practices (2019 Survey)

When pediatricians were asked to identify the methods utilized to generate referrals, those surveyed indicated that 51.8% of all patients are referred using electronic medical record (EMR). Providers were given the opportunity to ‘select all that apply’ so multiple methods for referral may have been identified for a single practice. On average, 1.84 modes for coordinating referrals are used in practice. However, 49.6% of practices use a single method, with EMR being the most common response (63.8% of practices that use a single method to generate referral rely on EMR) (Fig. 3).

**Fig. 4:**
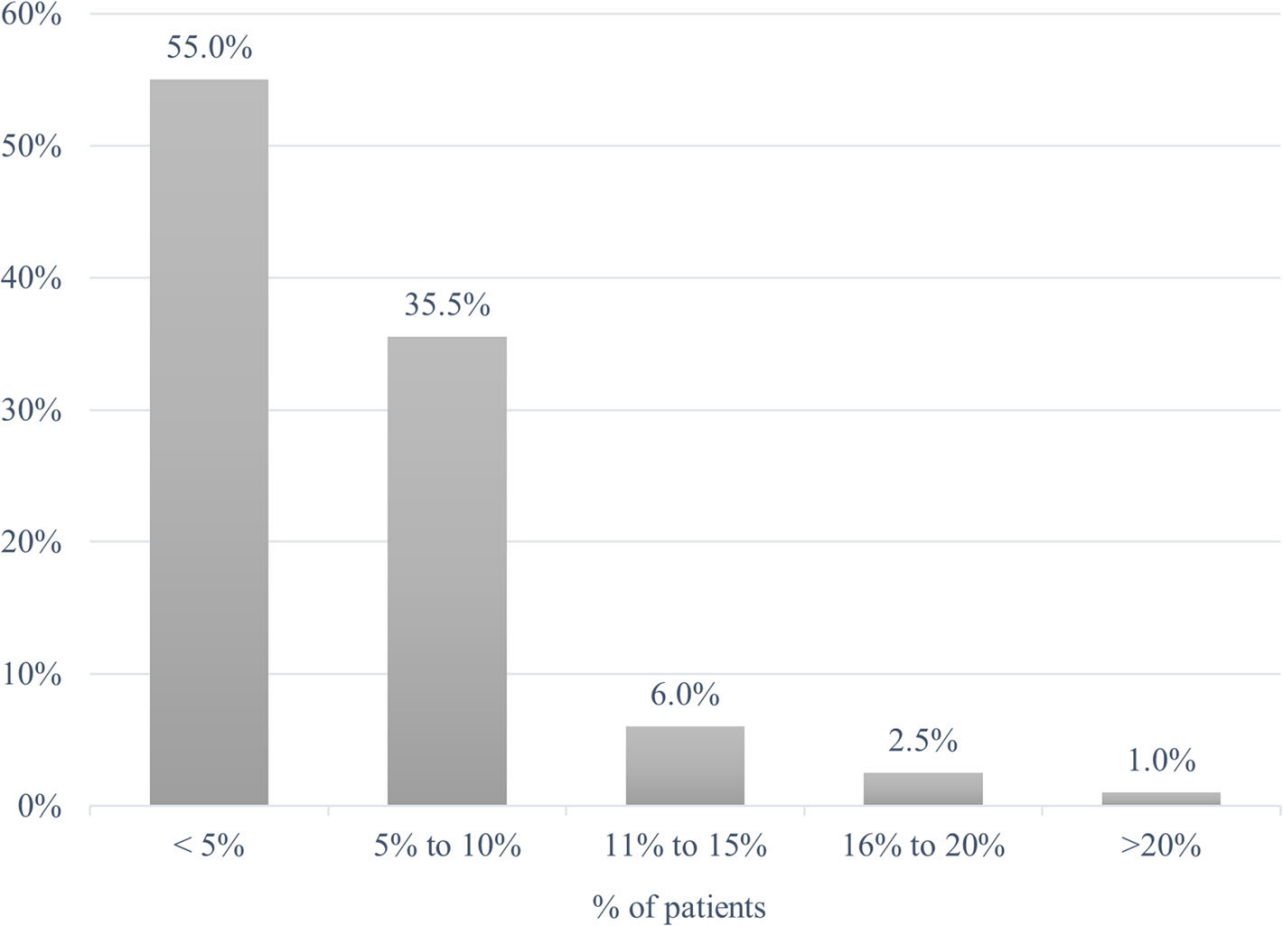
Percent of patients referred to a neurologist / pediatric neurologist in previous 12 months (2019 Survey)

In terms of how frequently pediatricians refer their patients for further evaluation to a neurologist / pediatric neurologist, 55.0% of pediatricians in 2019 said they have referred < 5% of patients over the prior 12 months, 35.5% reported referrals for 5 to 10% of patients, 6.0% noted referral for 11 to 15% of patients, and 2.5% indicated referrals for 16 to 20% of patients. One percent (1.0%) of pediatricians said they referred > 20% of patients to neurologists and/or pediatric neurologists for evaluation (Fig. 4).

Approximately two-thirds of respondents (*n = 390*) in 2019 said they had referred patients to a neurologist or pediatric neurologist once or twice for the evaluation of hypotonia in the prior year (32.2% selected ‘About once per year’ and 32.8% selected ‘About twice per year’). Of the remaining participants, 24.5% said they had made quarterly referrals while 10.5% reported monthly referrals.

**Fig. 5:**
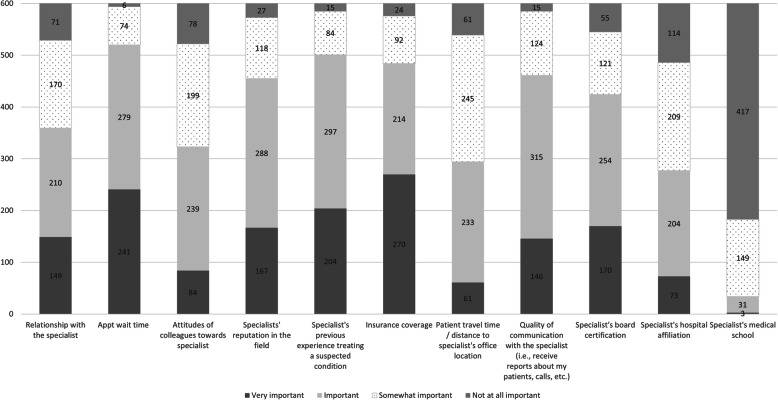
Factors considered when choosing a neurologist / pediatric neurologist for receipt of patient referral (2019 Survey)

In the 2019 study, participants were asked to rate the overall importance of different factors when selecting a neurologist or pediatric neurologist to refer a patient. The combined and ranked total number of factors identified by respondents as ‘Very important’ and ‘Important,’ identified the top 3 factors. Respondents identified appointment wait time (defined as the number of days between referral order and specialist appointment date) as the primary factor considered when selecting a neurologist or pediatric neurologist when generating a referral. The next factors most often cited were specialist’s previous experience treating a suspected condition, insurance coverage, quality of communication, and specialists’ reputation (Fig. 5 & Table 7).

**Table 7:** Factors considered ‘Very Important’ & ‘Important’ in choosing a neurologist / pediatric neurologist for referral (2019 Survey)

Factors Considered when Choosing a Neurologist /Pediatric Neurologist		% Respondents
1	Appointment wait time (i.e., wait time is the number of days between referral order and specialist appointment date)	86.7%
2	Specialist’s previous experience treating a suspected condition	83.5%
3	Insurance coverage	80.7%
4	Quality of communication with the specialist (i.e., receive reports about my patients, calls, etc.)	76.8%
5	Specialists’ reputation in his / her field, when known	75.8%

**Fig. 6:**
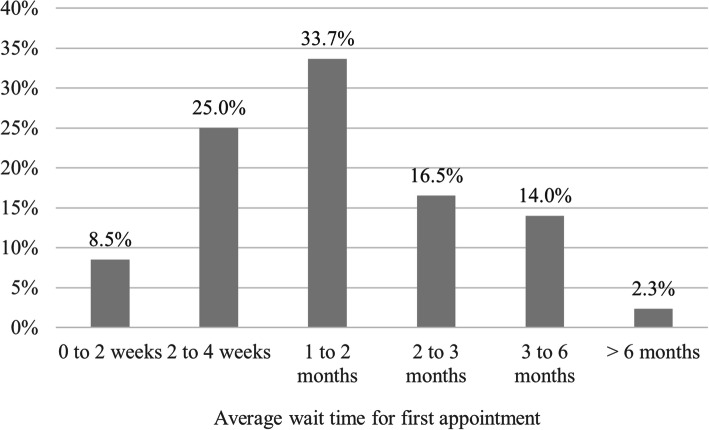
Average wait time for first appointment to see neurologists / pediatric neurologists in respondents’ region (2019 Survey)

When describing average wait times for first appointment with neurologist after referral, 64.2% of respondents in the 2019 survey indicated they had experienced wait times for specialist visits of 1–6 months, with most falling between a 1–2 month wait time (Fig. 6).

**Fig. 7:**
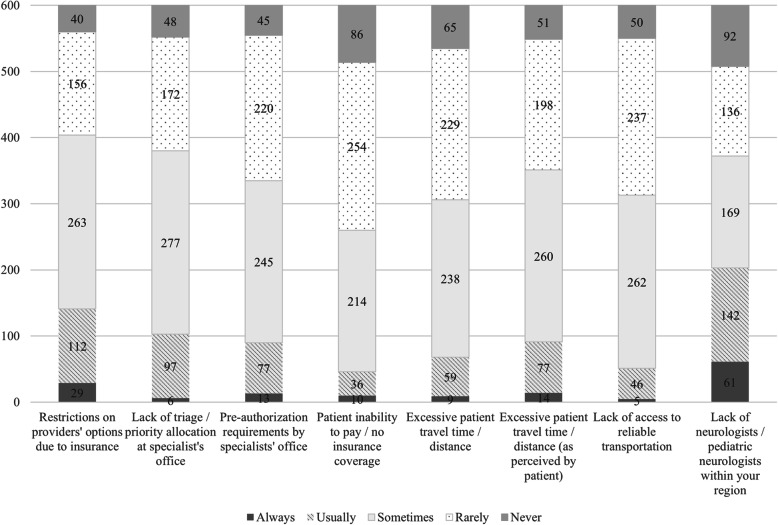
Perceived Contributors to Average Wait Time for Patients Completing a Neurology/Pediatric Neurology Referral (2019 Survey)

In ranking referral barriers perceived as contributors to lengthy appointment wait times, barriers that providers indicated ‘Always’, ‘Usually’ and ‘Sometimes’ were combined, yielding the top 3 reasons for extended appointment wait times as: restrictions due to insurance, lack of triage at specialist offices, and lack of neurologist or pediatric neurologist within the region (Fig. 7 & Table 8).

**Table 8:** Ranking of perceived referral barriers contributing to wait time (2019 Survey)

Always, Usually, & Sometimes		Rarely & Never	
Restrictions on providers’ options due to insurance	67.3%	Patient inability to pay / no insurance coverage	56.7%
Lack of triage / priority allocation at specialist’s office	63.3%	Excessive patient travel time / distance	49.0%
Lack of neurologists / pediatric neurologists within your region	62.0%	Lack of access to reliable transportation	47.8%

## Discussion

Despite the advent of genetic screening, a definitive tool for the diagnosis of SMA, enhanced education and awareness efforts regarding early symptoms, and three new FDA approved disease-modifying treatments, findings from these two surveys indicate continuing clinical knowledge gaps among pediatricians across sectors and experience levels, and point to potentially modifiable factors that contribute to the delay in SMA diagnosis. As evident by the findings of our studies and well-documented persistent diagnostic delay in SMA [31, 32], the need for increased awareness of the early signs and symptoms of SMA and the urgency of early treatment is clear.

Continuing education on the early symptoms of SMA, with an emphasis on the urgency to treat, will further enhance pediatrician awareness and inform clinical practice to ensure the best health outcomes for all children born with SMA. To promote reduction of diagnostic delays, the Cure SMA SMArt Moves education modules include a section specifically designed for health care professionals, which details current diagnostic criteria, educational resources, and the latest treatment options and protocols [41].

Early diagnosis of symptomatic SMA is prompted by the recognition of a cluster of physical signs and symptoms that are characteristic of the disease. Hallmark signs include progressive hypotonia and muscle weakness, areflexia, and motor delays and impairment in an alert, socially engaging child with normal cognition. Our surveys identified that a likely significant contributor to delayed diagnosis of SMA is the varied clinical response to the observation of hypotonia by pediatricians. While infant onset hypotonia is a frequent, nonspecific presenting symptom of an underlying NMD [30, 45], further evaluation of hypotonia leading to a confirmed diagnosis is frequently delayed for multiple reasons, including a large number of possible diagnoses, many of which are rare and often not treatable, perceived inconsistent symptom profile of cognitive and social alertness with motor weakness, and uncertainty about whether the observed or reported hypotonia is significant enough to warrant alarming parents [30].

Furthermore, these studies demonstrate significant variance in utilization of available screening tools among pediatricians. Since the early identification of developmental concerns leads to further evaluation of underlying etiology, pediatricians are encouraged to incorporate developmental screening within structured well visits [38, 39]. Current guidelines recommend the use of developmental screening tools at 9-, 18-, 30-, and 48-months, with ongoing surveillance at all remaining well visits and use of screening tools at the discretion of the provider and as elicited by parental concerns [39, 40]. However, surveillance alone has proven less effective than developmental screening tools to identify developmental delays [46–49]. Children screened using the developmental screening tools recommended by Bright Futures are more likely to receive a timely diagnosis and treatment than those receiving developmental surveillance alone. Physicians have a higher likelihood of recognizing early signs of delays, without the overidentification of false positives, when screening tools are utilized in clinical settings [46, 49–52]. Although genetic testing is required to diagnose SMA, consistent administration of developmental screening and surveillance may facilitate early recognition of concomitant physical signs in a symptomatic child, narrow the differential, and prompt further evaluation [29, 30].

Our findings are supported by an American Academy of Pediatrics membership survey assessing trends in use of developmental screening tools between 2002 and 2016 [53]. While significant progress was evident during that period, as 63% of pediatricians reported use of developmental screening tools in a 2016 survey as compared to only 21% in 2002, gaps in screening persist [53]. Efforts to advance further progress in adherence to screening guidelines include encouragement of pediatricians to utilize EMR and other tracking systems [53]. Additionally, to eliminate constraints to time, pediatricians may consider the recruitment of medical support and front office staff to distribute and score the tool as appropriate [53].

Additional barriers occur in the process of referring observed hypotonia to a specialist for further evaluation. While providers are encouraged to immediately refer the patient to a neurologist, pediatric neurologist, or neuromuscular specialist for evaluation and genetic testing, a critical barrier is lengthy wait times for a specialist appointment, for patients with symptoms of NMD and possible SMA. Given what is known about SMA and the importance of early diagnosis and early treatment, appointment wait times of 1–6 months that were cited by 64.2% of survey respondents, will delay access to a disease-modifying early intervention that may significantly alter their prognosis.

Given that SMA is a rare disease, there is an opportunity to join forces with others in the pediatric rare NMD community to advance solutions that could benefit patients across the NMD spectrum facing similar diagnostic delays, including Pompe [54] and Duchenne Muscular Dystrophy [55]. Findings from our SMA studies provide additional evidence to support collaborative efforts to reduce diagnostic delays by advancing policies to expand newborn screening to all states, enhance insurance coverage and broaden access to specialists, partner with specialists and pediatricians to improve communication, and develop additional guidelines for developmental screening, genetic testing and triage guidelines to expedite referrals for children with pediatric hypotonia and motor delay.

As motor neurons are lost over time with SMA, there is an imperative to accelerate the process of confirming diagnosis and accessing interventions. Newborn screening facilitates early diagnosis and presymptomatic treatment. The current treatment algorithm for infants diagnosed via newborn screening offer guidance for providers regarding the appropriateness of treatment initiation and surveillance based on *SMN2* copy number [56, 57]. However even in states in which SMA screening has been implemented, providers should remain vigilant as 3 to 5% of individuals with SMA will not be identified due to *SMN1* point mutations. Upon recognition of the early signs of NMD, pediatric primary care providers are encouraged to immediately refer patients to a neurologist or neuromuscular specialist for further evaluation, including genetic testing which has been validated to provide a definitive means of diagnosis for SMA [24]. Effective communication via the provision of quality and timely referrals provides the opportunity to efficiently coordinate care while promoting accessibility to specialty care [58–61].

### Study limitations

Due to the method of recruitment, there is a sampling bias within our research design. Medscape maintains a robust database of providers within the United States. However, we recognize that our sample does not fully represent the full population of general pediatricians as providers must independently create an account to access content on Medscape’s platform. Also, providers with an interest in receiving market research invitations from Medscape are required to complete an additional opt-in process while logged-in to their perspective accounts. Additionally, to achieve the target number of responses, invitations for the 2018 and 2019 surveys were distributed via a batch method to eligible providers. Given the method for distribution, a lower response rate was obtained for each survey (Table 4).

## Conclusions

The learnings from these studies will continue to inform efforts to reduce diagnostic delay and alleviate barriers to optimal diagnosis and management of SMA. As follow up to this work, Cure SMA will conduct a survey for SMA specialists and neurologists, to identify best practices to triage referrals for the evaluation of hypotonia while further examining the average wait time experienced by individuals and families affected by SMA. It is vital to continue to leverage new discoveries and knowledge about the early signs of SMA, ensuring the earliest possible diagnosis and intervention by pediatricians, pediatric neurologists and other NMD specialists. Cure SMA seeks to continue partnerships with all stakeholders – clinicians, industry, policy makers and members of the SMA community to achieve a near-term future when all SMA patients are promptly diagnosed and receive appropriate intervention to ensure their best possible outcomes.

## Supplementary Information

**Additional file 1.**

## Data Availability

The data collected and analyzed during the current study is generated and owned by Cure SMA and not publicly available. However, Cure SMA will consider for the provision of the raw data set upon reasonable request.
