# Supplementary material for: Awareness screening and referral patterns among pediatricians in the United States related to early clinical features of spinal muscular atrophy (SMA)
Source: BMC Pediatr. 2021 May 17;21:236. doi: 10.1186/s12887-021-02692-2 (PMC8127310; doi:10.1186/s12887-021-02692-2)
Supplement: Supplementary file 1 — Additional file 1. [file 12887_2021_2692_MOESM1_ESM.docx]

**Appendix 1: Cure SMA 2018 General Pediatrician Survey**

Thank you for your participation in this brief survey. To improve newborn screening follow-up, and facilitate early diagnosis in children born prior to your state’s adoption of SMA into their Newborn Screening panel, Cure SMA is working to create reference materials and diagnostic toolkits. By completing this brief survey, you will have a direct impact on the development of future resources.

All personal information and individual responses will be kept confidential.  Aggregate data may be shared with Cure SMA grant recipients, content providers, sponsors, service providers or other third parties to advance the goals of research, education, dissemination of information and otherwise in fulfillment of the goals of Cure SMA. Cure SMA maintains commercially reasonable safeguards to ensure the security, integrity and privacy of the Personal Information provided by you. For more information please visit Cure SMA’s [privacy policy page.](http://www.curesma.org/privacy-policy.html)

The survey itself will take approximately 10 minutes to complete. If you have any questions or concerns, please feel free to contact Mary Curry at [mary.curry@curesma.org](mailto:mary.curry@curesma.org).

1. Specialty:
   1. General Pediatrics
   2. Developmental Pediatrics
   3. Emergency Medicine
   4. Geneticist
   5. Neonatology
   6. Neurology
   7. Nurse Practitioner
   8. Pediatric Critical Care
   9. Pediatric Neurology
   10. Pediatric Pulmonology
   11. Other: Click or tap here to enter text.
2. How many years have you been in practice?
3. 0 to 5 years
4. 6 to 10 years
5. 11 to 20 years
6. 21 to 30 years
7. Over 30 years
8. What developmental screening tools are utilized in your practice? (Select all that apply)
   1. Ages and Stages Questionnaire (ASQ-3)
   2. Battelle Developmental Inventory Screening (BDI-ST)
   3. Bayley Infant Neurodevelopmental Screen (BINS)
   4. Brigance Screens-II
   5. Child Development Inventory (CDI)
   6. Child Development Review-Parent Questionnaire (CDR-PQ)
   7. Denver-II Developmental Screening Test
   8. Infant Development Inventory
   9. Parents’ Evaluation of Developmental Status (PEDS)
   10. Other Click or tap here to enter text.
9. How frequently are developmental screening tools administered? (Select all that apply)
   1. Tools are administered at each well visit
   2. Tools are administered at 9-, 18-, and 30-month visits
   3. Tools are administered as concerns appear during developmental surveillance
10. When you observe hypotonia in an infant or toddler, what is your typical course of action? (select all that apply)
    1. Wait and see, evaluate at next scheduled wellness visit
    2. Schedule an early return visit within a month
    3. Prompt immediate referral to pediatric neurologist for further evaluation
    4. Prompt immediate referral to early intervention
    5. Order Serum Creatine Kinase test
    6. Other (explain) Click or tap here to enter text.
11. When suspecting motor delay, how often do you refer a child to a specialist (e.g., pediatric neurologist) for further evaluation?
    1. Very infrequently
    2. Somewhat infrequently
    3. Occasionally
    4. Somewhat frequently
    5. Very frequently
12. I feel comfortable identifying the early signs and symptoms of neuromuscular disorders.
    1. Not at all comfortable
    2. Slightly comfortable
    3. Moderately comfortable
    4. Very comfortable
    5. Extremely comfortable
13. Please rate your current familiarity with Spinal Muscular Atrophy (SMA):
    1. Not at all familiar
    2. Slightly familiar
    3. Moderately familiar
    4. Very familiar
    5. Extremely familiar
14. Which of the following procedures is **required** to make a definitive diagnosis of Spinal Muscular Atrophy (SMA)?
    1. Electromyography
    2. Genetic Testing
    3. Muscle Biopsy
    4. Serum Creatine Kinase
    5. Other
15. Indicate whether each statement listed below is True or False.

|  | **True** | **False** |
| --- | --- | --- |
| Lack of spontaneous movement at well visit warrants immediate referral to pediatric neurologist for evaluation. |  |  |
| Upon suspicion of motor delay, physical examination should include observation of age-appropriate motor skills such as pull-to-sit, sitting, rising to stand, and walking/running. |  |  |
| Normal serum creatine kinase rules out neuromuscular disorders. |  |  |
| The efficacy of the only FDA approved therapy for SMA depends on the timing of administration (the earlier the exposure the greater the chances for improvement in achieved milestones). |  |  |

1. Please identify what educational sources (information, materials) would help you to more readily identify the early symptoms of Spinal Muscular Atrophy (SMA) in an infant or young child? (select all that apply)
   1. CME
   2. Reference Materials
   3. Short videos featuring tools for evaluation
   4. Short videos reviewing early clinical presentation of SMA in infants
   5. Toolkit
   6. Webinar
   7. Other Click or tap here to enter text.

**Appendix 2: Cure SMA 2019 General Pediatrician Referral Survey**

Thank you for your participation in this brief survey. [Cure SMA](https://www.curesma.org/) is the leading non-profit patient advocacy organization dedicated to the treatment and cure of spinal muscular atrophy (SMA) – the number one genetic cause of death for infants. **This** **survey seeks to examine the factors that impact current referral practices by general pediatricians**. By completing this brief survey, you will help to inform the development of future resources to address barriers to diagnosis and treatment upon suspicion of neurological disease and observation of hypotonia.

All personal information and individual responses will be kept confidential. Aggregate data may be shared with Cure SMA grant recipients, content providers, sponsors, service providers, or other third parties to advance the goals of research, education, dissemination of information, and otherwise in fulfillment of the goals of Cure SMA. Cure SMA maintains commercially reasonable safeguards to ensure the security, integrity, and privacy of the Personal Information provided by you. For more information please visit Cure SMA’s [privacy policy page.](http://www.curesma.org/privacy-policy.html)

The survey itself will take approximately 15 to 25 minutes to complete. If you have any questions or concerns, please feel free to contact Mary Curry at [mary.curry@curesma.org](mailto:mary.curry@curesma.org).

**Demographics**

1. What is your specialty?
2. General Pediatrics
3. Developmental Pediatrics (END SURVEY)
4. Emergency Medicine (END SURVEY)
5. Geneticist (END SURVEY)
6. Neonatology (END SURVEY)
7. Neurology (END SURVEY)
8. Pediatric Critical Care (END SURVEY)
9. Pediatric Neurology (END SURVEY)
10. Pediatric Pulmonology (END SURVEY)
11. Other: Click or tap here to enter text. (END SURVEY)
12. How many years have you been in practice?
13. 0 to 10 years
14. 11 to 20 years
15. 21 to 30 years
16. Over 30 years
17. On average, how many patients do you see each week?
18. 0 to 10
19. 11 to 25
20. 26 to 50
21. 51 to 75
22. >75
23. Where is your practice located?
24. Urban
25. Rural
26. Suburban
27. What is the zip code for the practice location in which you spend the majority of your time? (TEXT BOX)
28. Type of practice?
29. Solo practice (SKIP NEXT QUESTION)
30. Single specialty group
31. Multi-specialty group
32. Direct hospital employee/contractor
33. Academic faculty practice
34. Other (TEXT BOX)
35. How many physicians are in in your practice?
36. Less than 5
37. 5 to 10
38. 11 to 24
39. 25 to 49
40. 50+
41. Please indicate the number of managed care contracts.
42. 0
43. 1 to 4
44. 5 to 9
45. 10+

**Questions on SMA Awareness and Developmental Screening**

1. What developmental screening tools are utilized in your practice? *(Select all that apply)*
   1. Ages and Stages Questionnaire (ASQ-3)
   2. Battelle Developmental Inventory Screening (BDI-ST)
   3. Bayley Infant Neurodevelopmental Screen (BINS)
   4. Brigance Screens-II
   5. Child Development Inventory (CDI)
   6. Child Development Review-Parent Questionnaire (CDR-PQ)
   7. Denver-II Developmental Screening Test
   8. Infant Development Inventory
   9. Parents’ Evaluation of Developmental Status (PEDS)
   10. Other (TEXT BOX)
2. How frequently are developmental screening tools administered within your practice?
   1. I do not utilize developmental screening tools in practice
   2. Tools are administered at 9-, 18-, and 30-month visits
   3. Tools are administered as concerns appear during developmental surveillance
   4. Tools are administered at 9-, 18-, and 30-month visits and as concerns appear during developmental surveillance
   5. Tools are administered at each well visit
3. Which of the following procedures is required to make a definitive diagnosis of Spinal Muscular Atrophy (SMA)?
4. Electromyography
5. Genetic Testing
6. Muscle Biopsy
7. Serum Creatine Kinase
8. Other (TEXT BOX)

**Questions on Referral Process**

1. Please select the modes by which referrals are coordinated in your practice. *(Select all that apply)*
2. Referral sent via EMR
3. Referral sent via fax
4. Referral made via phone
5. Referral made via secure email
6. Referral submitted via centralized intake
7. Other (TEXT BOX)
8. Based on your selections above, please indicate the percent (%) of patients referred via each method.
9. Referral sent via EMR: (TEXT BOX)
10. Referral sent via fax: (TEXT BOX)
11. Referral made via phone: (TEXT BOX)
12. Referral made via secure email: (TEXT BOX)
13. Referral submitted via centralized intake: (TEXT BOX)
14. Other: (TEXT BOX)
15. Do you utilize a standardized referral form?
16. Yes
17. No (SKIP TO QUESTION 13)
18. **If yes**, which of the following applies?
    1. My practice and/or hospital generated the standardized form and it is utilized for all my outgoing referrals.
    2. I utilize the standardized referral form that was generated and posted by the hospital affiliated with the specialist of choice.
    3. I utilize a standardized referral form that was generated and posted by a hospital within my region.
    4. I utilize a standardized referral form that was generated and posted by a professional organization (i.e., AAP, AAN, etc.).
    5. Other (TEXT BOX)
19. In the past 12 months, how frequently have you provided information regarding your patient’s history and reason for consult, to the specialist, when generating a referral?
20. Always
21. Usually
22. Sometimes
23. Rarely
24. Never
25. In the past 12 months, how frequently have you received a referral acknowledgement, from a specialist, regarding a patient that you have referred?
26. Always
27. Usually
28. Sometimes
29. Rarely
30. Never
31. In your experience, what is the average turnaround time for a referral acknowledgment to be received from a specialist’s office, following the provision of a referral?
    1. Same day
    2. 1 business day
    3. 2 business days
    4. 3 to 5 business days
    5. >5 business days
    6. Not Applicable – Referral acknowledgement not received prior to patients’ appointment with specialist
32. In the past 12 months, how frequently have you received a report that includes exam / test results and treatment plans (when appropriate), from a specialist, regarding a patient that you have referred?
33. Always
34. Usually
35. Sometimes
36. Rarely
37. Never
38. In your experience, what is the average wait time for a first appointment to see neurologists / pediatric neurologists within your region? *(Wait time is defined as the number of days between referral order and specialist appointment date)*
39. 0 to 2 weeks
40. 2 to 4 weeks
41. 1 to 2 months
42. 2 to 3 months
43. 3 to 6 months
44. < 6 months
45. In your opinion, how often do the following referral barriers contribute to the current wait time of your patients seeking to complete a neurology / pediatric neurology referral? (*Please rate each barrier based on the following scale: Always, Usually, Sometimes, Rarely, Never)*

|  | Always | Usually | Sometimes | Rarely | Never |
| --- | --- | --- | --- | --- | --- |
| Restrictions on providers’ options due to insurance |  |  |  |  |  |
| Lack of triage / priority allocation at specialist’s office |  |  |  |  |  |
| Pre-authorization requirements by specialists’ office |  |  |  |  |  |
| Patient inability to pay / no insurance coverage |  |  |  |  |  |
| Excessive patient travel time / distance |  |  |  |  |  |
| Excessive patient travel time / distance *(as perceived by patient)* |  |  |  |  |  |
| Lack of access to reliable transportation |  |  |  |  |  |
| Lack of neurologists / pediatric neurologists within your region |  |  |  |  |  |
| Other (TEXT BOX) / please rate |  |  |  |  |  |

1. In the past 12 months, what percentage (%) of your patients were referred to a neurologist / pediatric neurologist for further evaluation?
2. < 5%
3. 5% to 10%
4. 11% to 12%
5. 16% to 20%
6. >20%
7. In the past 12 months, approximately how frequently did you refer a pediatric patient to a neurologist / pediatric neurologist for the evaluation of hypotonia?
8. Monthly
9. Quarterly
10. About twice a year
11. About once a year
12. Please rate the overall level of importance of the following factors when choosing a neurologist / pediatric neurologist, to whom to refer your patient. (*Please rate each factor using the following scale: Very important, Important, Somewhat important, not at all important)*

|  | Very important | Important | Somewhat important | Not at all important |
| --- | --- | --- | --- | --- |
| Your relationship with the specialist |  |  |  |  |
| Appointment wait time (*i.e., wait time is the number of days between referral order and specialist appointment date)* |  |  |  |  |
| Attitudes of colleagues towards specialist |  |  |  |  |
| Specialists’ reputation in his / her field, when known |  |  |  |  |
| Specialist’s previous experience treating a suspected condition |  |  |  |  |
| Insurance coverage |  |  |  |  |
| Patient travel time/distance to specialist’s office location |  |  |  |  |
| Quality of communication with the specialist *(i.e., receive reports about my patients, calls, etc.)* |  |  |  |  |
| Specialist’s board certification |  |  |  |  |
| Specialist’s hospital affiliation |  |  |  |  |
| Specialist’s medical school |  |  |  |  |

1. Does your practice designate specific staff to coordinate patient care with specialists?

Yes

No

1. Does your staff track referral status (*i.e. specialist has reviewed and approved / denied a referral, patient has scheduled appointment to see specialist, specialist appointment has been completed*)?

Yes

No

Unknown

1. Are scheduling rates for outgoing referrals tracked within your electronic medical records (EMR)?

Yes

No

Unknown
